# Conscious Sedation versus General Anesthesia for Patients with Acute Ischemic Stroke Undergoing Endovascular Therapy: A Systematic Review and Meta-Analysis

**DOI:** 10.1155/2018/2318489

**Published:** 2018-03-29

**Authors:** Ren Jing, Hui-jun Dai, Fei Lin, Wan-yun Ge, Ling-hui Pan

**Affiliations:** ^1^Department of Anesthesiology, Tumor Hospital of Guangxi Medical University, Nanning, Guangxi 530021, China; ^2^The Laboratory of Perioperative Medicine Research Center, Tumor Hospital of Guangxi Medical University, Nanning, Guangxi 530021, China

## Abstract

The aim of this study is to compare the effect of conscious sedation (CS) with general anesthesia (GA) on clinical outcomes in patients with acute ischemic stroke (AIS) undergoing endovascular therapy (EVT). MEDLINE, EMBASE, and Cochrane Central Registers of Controlled Trials (from inception to July 2017) were searched for reports on CS and GA of AIS undergoing EVT. Two reviewers assessed the eligibility of the identified studies and extracted data. Data were analyzed using the fixed-effects model, and the sources of heterogeneity were explored by sensitive analysis. Trial sequential analysis was conducted to monitor boundaries for the limitation of global type I error, and GRADE system was demonstrated to evaluate the quality of evidence. A total of thirteen studies were finally identified. Pooled analysis of the incidence of mRS score ≦ 2 after hospital discharge and one or three months in the CS group was higher than that in the GA group. The all-causing mortality of AIS patients in the CS group was lower than that in the GA group. There were no differences in the proportion of IA rtPA and thrombolysis between the two groups. Compared with AIS patients receiving GA, the all-causing mortality in the AIS patients receiving CS was decreased, while incidence of mRS score ≦ 2 at hospital discharge and one or three months was increased.

## 1. Introduction

Acute ischemic stroke (AIS), a medical condition due to low blood flow to the brain, is the third-leading cause of death and induced by thrombosis, embolism, systemic hypoperfusion, and cerebral venous sinus thrombosis [1–3]. Although approximately 120 million neurons are dying each hour, definitive therapy is concentrated on elimination of the blockage by thrombolysis or mechanical thrombectomy within the first few hours [4]. Endovascular therapy (EVT) included intra-arterial (IA) thrombolysis by recombinant tissue plasminogen activator (rtPA) and mechanical embolectomy, which has been reported to be the satisfactory impact on vessel recanalization and revascularization of patients with AIS [5–7].

Clinical outcomes of stroke are associated with some factors such as blood pressure, blood glucose, fluid, and temperature management. Recently, type of anesthesia on the clinical outcomes during EVT for patients with AIS has attracted many researchers' attention. One study reported that interventions for patients with anterior circulation stroke under general anesthesia (GA) were ranging from 0% to 100% and the average was 44% among centers [8]. The safety of AIS patients under GA for IA therapy has been performed, but recent data were still controversial [9, 10]. However, conscious sedation (CS) without intubation for EVT in AIS patients would decrease the risk of aspiration and potential airway injury by intubation, prevent cardiovascular and pulmonary complications, and allow operators to monitor neurologic status during the procedure.

There was no sufficient evidence to suggest that CS is a better type of anesthesia on the clinical outcomes of AIS patients undergoing EVT in comparison with GA. A study by Brinjikji et al. in 2015 reported that AIS patients undergoing IA therapy received GA resulting in worse outcomes compared with CS but the difference in stroke severity at the onset confounded the comparison [11]. Hence, we sought to conduct a systematic review and meta-analysis to compare the impact of CS with GA for patients with AIS undergoing EVT by identified retrospective studies or randomized controlled trials (RCT) from inception to July 2017.

## 2. Materials and Methods

### 2.1. Study Search

The Preferred Reporting Items for Systematic Reviews and Meta-analyses statement and recommendations of the Cochrane handbook for systematic reviews of interventions were used during the design, implementation, and reporting of this study [22, 23]. The electronic databases, including MEDLINE, EMBASE, and Cochrane Central Registers of Controlled Trials (from inception to July 2017), were searched for reports of articles pertaining to the CS versus GA for AIS patients under EVT. The search terms used were* “conscious sedation”*,* “general anesthesia”*, and* “stroke”*, and the concerned treatment techniques included endovascular thrombolysis, fibrinolysis, thrombectomy, fibrinolytic agents, recanalization, embolectomy by catheter or transcatheter. Additional references were manually searched from multiple articles or bibliographies. These citations were included in this meta-analysis.

The included studies from the study search that compared angiographic and clinical outcomes between the GA/intubated anesthesia and CS/nonintubated anesthesia/local anesthesia group for AIS patients under EVT were identified in the present meta-analysis. Exclusion criteria were the following: (1) case reports or review; (2) studies not separating outcomes by anesthesia type; (3) noncomparative studies (i.e., studies with only CS or GA). RJ and H-J D reviewed the articles for inclusion, and the data were abstracted by RJ. Disagreements were resolved by discussion with other authors.

### 2.2. Quality Assessment

Newcastle-Ottawa scale, a common tool for assessing the quality of nonrandomized studies included in systematic reviews and/or meta-analyses, was used for included studies in the present study. Each study is evaluated on eight items categorized into selection of the study groups, comparability of the study groups, and ascertainment of the outcome of interest. One plus indicates each quality item; four pluses are the maximum for the selection; two pluses are the maximum for the comparability; three pluses are the maximum for completeness of the outcomes. The highest quality studies are awarded up to nine pluses.

### 2.3. Outcome Variables

The primary outcome was modified Rankin Scale (mRS) score of ≦2 at discharge and one or three months following EVT. The second outcomes included all-cause mortality, percentage of IA rtPA, and thrombolysis during EVT. For each study, mRS score of ≦2 and mortality were determined at hospital discharge and one or three months of follow-up. Furthermore, we also collected the following relevant information: type of study, type of stroke, type of EVT, and sample size of CS or GA group for each included study.

### 2.4. Statistics

The odds ratios (OR) and their respective 95% confidence intervals (95% CI) for the binary outcomes of each study were presented. The Mantel-Haenszel fixed effect's model was primarily used while Mantel-Haenszel random effects model was performed if heterogeneity > 50%. The statistical heterogeneity across studies was assessed using the Cochrane's chi-squared test. The Higgins' inconsistency test (*I*^2^) was performed to quantify the percentage of the variability in the estimated effect due to heterogeneity rather than chance. *I*^2^≦25% indicated low heterogeneity and ≧75% defined as high heterogeneity. While the heterogeneity was bigger than 50% with *P* < 0.05 in the pooled outcomes, sensitivity analysis and metaregression were performed to explore the source of heterogeneity. The probability of publication bias was analyzed by visual inspection of funnel plot and considered plot asymmetry that was tested by Begg's test and Egger's test to be suggestive of reporting bias. All analyses were performed using the Stata version 11.0 (StataCorp, College Station, TX).

To calculate the optimal event size requirement for our study, a formal trial sequential analysis (TSA; TSA software version 0.9 Beta; Copenhagen Trial Unit, Copenhagen, Denmark) was performed considering a mortality rate of 3% in the control group, a relative risk reduction of 35%, 80% of power, and a type I error of 5%. Furthermore, the Grading of Recommendations Assessment, Development, and Evaluation (GRADE) system was used to assess the certainty of evidence for each outcome [24]. Certainty of evidence considers the participants (studies); median follow-up; risk of bias, precision, directness, and consistency of the evidence; the probability of publication bias.

## 3. Results

### 3.1. Literature Search

**Figure 1:**
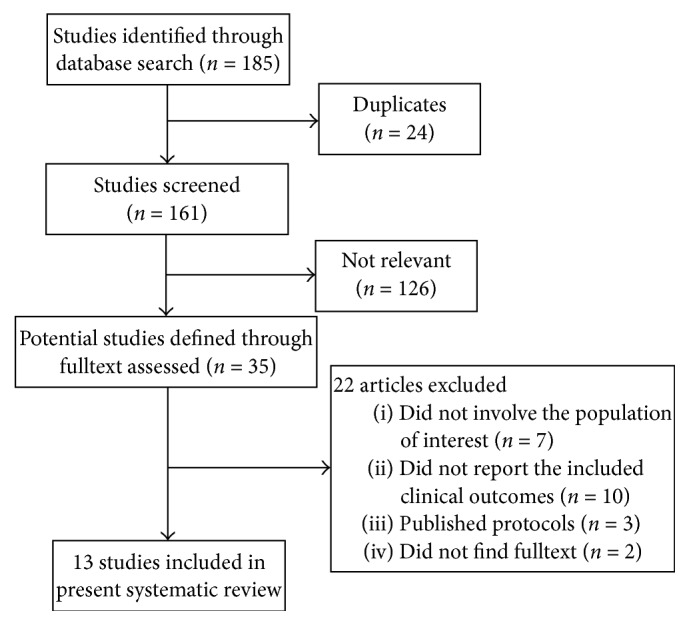
Study search and selection processes.

A total of 185 articles were identified with the subject heading's* conscious sedation, general anesthesia, *and* stroke *(Figure 1). The language and species of potential articles were limited to English and humans, respectively, and publish dates of searched articles were between 1985 and 2017, which yielded 150 (81.1%) articles. Then, the remaining articles were manually searched and 13 (7.0%) studies [8–10, 12–21] that met the prespecified criteria were finally identified. Among the 22 excluded articles, 7 (3.8%) articles did not involve the population of interest, 10 (5.4%) articles did not report the included clinical outcomes, 3 (1.6%) articles were published protocol, and 2 (1.1) did not find the full text.

The included studies compared 1443 patients in CS group and 1205 patients in GA group. The largest study [13] had 647 patients (387 with CS and 260 with GA), and the smallest study had 35 patients (18 with CS and 17 with GA). Majority of included references [8, 9, 12–17, 20, 21] were retrospective analysis except for two randomized controlled trials [10, 18] and one pilot study [19]. The included studies were both undergoing EVT such as solitaire stent, IA rtPA, recanalization, mechanical thrombectomy, thromboaspiration, or thrombus aspiration. All studies had at least five pluses on the Newcastle-Ottawa scale. A summary of included studies is shown in Table 1.

### 3.2. Publication Bias and GRADE Evidence Profile

**Figure 2:**
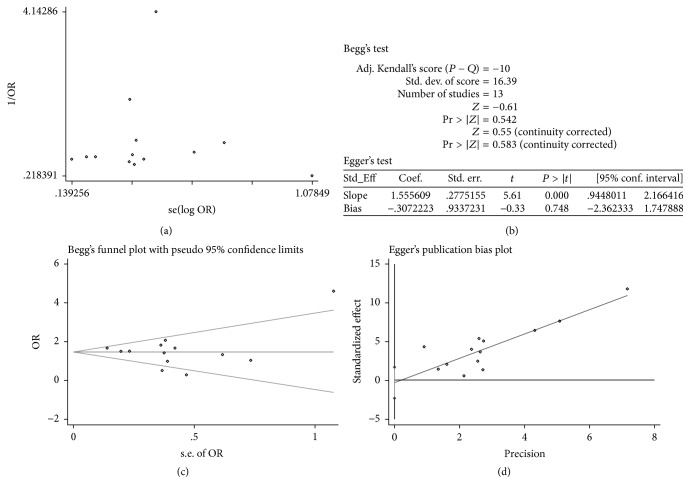
Publication bias from the data of primary outcome. (a) Funnel plot; (b) data of Begg's and Egger's test; (c) Begg's funnel plot; (d) Egger's publication bias plot. OR: odds ratio.

In this study, the majority of included studies were within the confidence limit except for one study in funnel plot (Figure 2(a)), suggesting that basically there was no publication bias detected. Simultaneously, the *P* values of Begg's test and Egger's test were 0.583 and 0.748, respectively (Figures 2(b)–2(d)), being consistent with the result of funnel plot. The limitation between these test methods might be derived from small sizes of this study or the amount of included studies. As Table 2 showed that the incidence of mRS score ≦ 2, all-causing mortality and incidence of IA rtPA were both defined as high-quality evidence, and the incidence of thrombolysis was moderate- quality evidence according to the GRADE evidence profile.

### 3.3. Primary Outcomes

**Figure 3:**
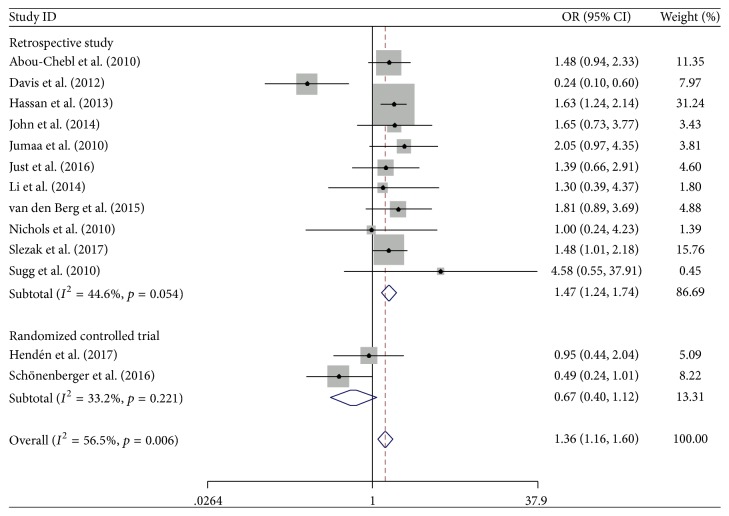
Forest plot of the incidence of mRS ≦ 2 after three months. OR: odds ratio; 95% CI: 95% confidence interval.

**Figure 4:**
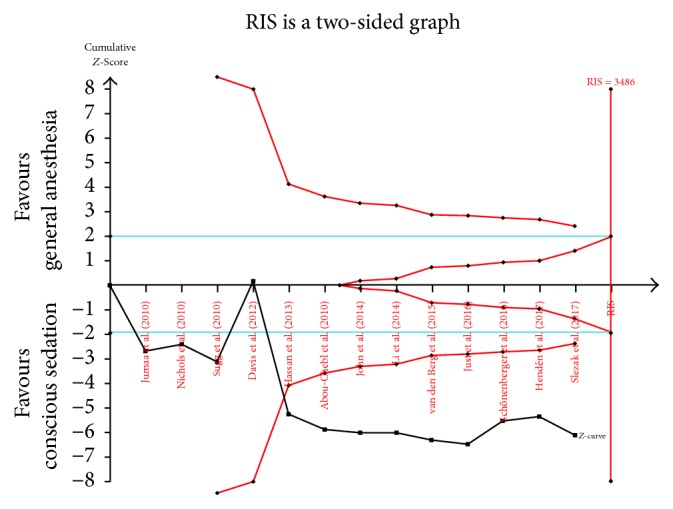
TSA graph of the incidence of mRS ≦ 2 after three months. RIS: required information size.

Thirteen trials [8–10, 12–21] were included in our meta-analysis assessing the effect of CS versus GA on the incidence of mRS score ≦ 2 at hospital discharge and one or three months in patients with AIS (Figure 3). This outcome was divided into two subgroups according to the study design (retrospective study or RCT). In the subgroup analysis of retrospective study, the incidence of mRS score ≦ 2 at hospital discharge and one or three months was 44.1% (582/1319) in the CS group and 32.3% (348/1079) in the GA group (OR 1.47; 95% CI 1.24–1.74; *I*^2^ = 44.6%). In the subgroup analysis of RCTs, the incidence of mRS score ≦ 2 at hospital discharge and one or three months was 26.3% (32/122) in the CS group and 39.0% (46/118) in the GA group (OR 0.67; 95% CI 0.40–1.12; *I*^2^ = 33.2%). Pooled analysis of the incidence of mRS score ≦ 2 at hospital discharge and one or three months in the CS group was higher than that in the GA group (OR 1.36; 95% CI 1.16–1.60; *I*^2^ = 56.5%). However, the total number of mRS scores ≦ 2 at hospital discharge and one or three months was 1008, which is less than the optimal event size (3486 events); that is, the TSA indicated an overall type I error of 5% for the meta-analysis result. In this case, the TSA graph shows that the cumulative *Z* curve does not exceed the TSA boundary line and is, therefore, insufficient to demonstrate that the test group CS is indeed superior to GA in terms of AIS (Figure 4).

### 3.4. Secondary Outcomes

**Figure 5:**
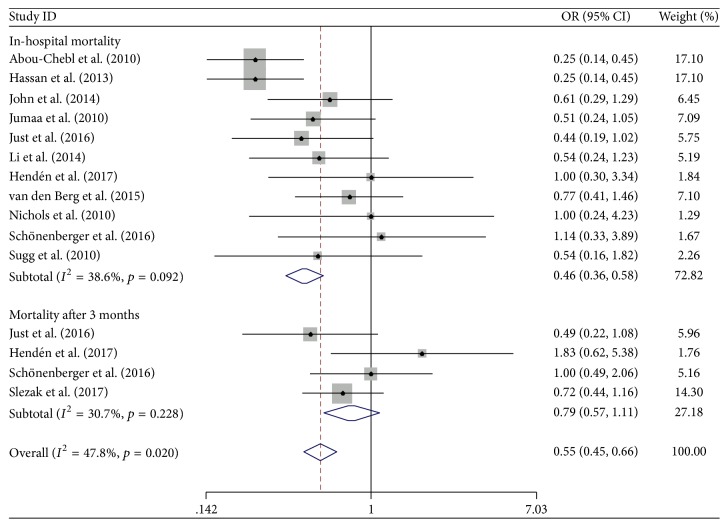
Forest plot of the all-causing mortality in hospital and after three months. OR: odds ratio; 95% CI: 95% confidence interval.

**Figure 6:**
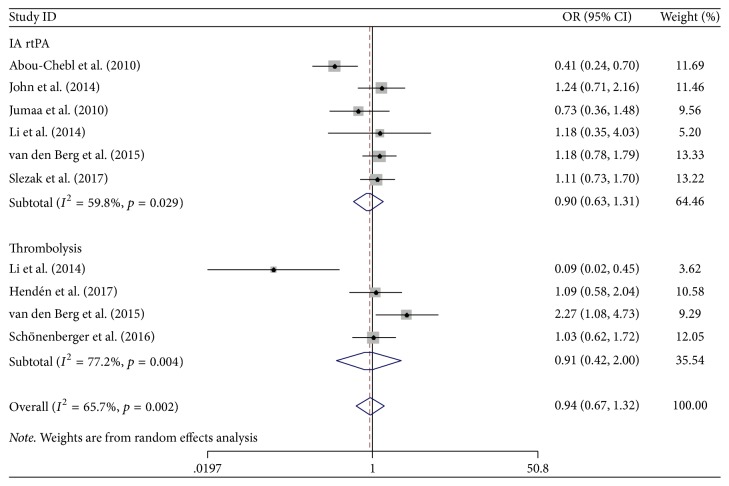
Forest plot of the utilization rates of IA rtPA and thrombolysis in the hospital. OR: odds ratio; 95% CI: 95% confidence interval.

The effects of CS and GA on secondary outcomes are presented in Figures 5 and 6. CS was associated with a decreased risk of in-hospital mortality (OR 0.46; 95% CI 0.36–0.58; *I*^2^ = 38.6%) in comparison with GA. However, there was no difference in the mortality after three months between groups (OR 0.79; 95% CI 0.57–1.11; *I*^2^ = 30.7%). The pooled analysis of all-causing mortality showed that the all-causing mortality of AIS patients in the CS group was lower than that in the GA group (OR 0.55; 95% CI 0.45–0.66; *I*^2^ = 47.8%) (Figure 5). Furthermore, there were no differences in the incidence of IA rtPA and thrombolysis in the hospital between the two groups (Figure 6).

## 4. Discussion

Our analysis of moderate-quality evidence showed that CS reduces all-causing mortality but increases the incidence of mRS score ≦ 2 at three months among patients with AIS receiving EVT, and there were no differences in the incidence of IA rtPA and thrombolysis in the hospital between the two groups. These data suggested that CS could be an anesthesia technique of choice during EVT of AIS patients.

National Institutes of Health Stroke Scale (NIHSS) score was used to assess the severity of AIS in the all included studies. Seven studies [9, 10, 14, 16, 17, 19, 21] showed that the statistical difference of NIHSS score at admission between the CS and GA groups was not found, and six studies [8, 12, 13, 15, 18, 20] reported that the mean admission NIHSS score in patients under CS was lower than that in the patients who were treated under GA from initiation. The patients with severe AIS by higher NIHSS score were more vulnerable to treat under GA, which may be contributed to the heterogeneity of analyzed outcomes. In the all included studies, there were no statistical difference in the location of stroke between the CS and GA groups and no description of the posterior circulation stroke.

In 2013, approximately 6.9 million people had an ischemic stroke, and there were about 3.0 million deaths that resulted from ischemic stroke [25, 26]. AIS is generally caused by blockage of a blood vessel resulting from the abscission of mural thrombus on the inner wall of brain blood vessels, though there are also fewer common causes. It is more vulnerable to mural thrombosis in the heart valve of those patients who suffer from coronary heart disease with atrial fibrillation [27]. Hypertension is reported as the most important risk factor for AIS, especially abnormal increase of blood pressure in the morning. It is reported that the risk of ischemic stroke in the early morning period is four times that of other periods, and every morning blood pressure increases by 10 mmHg and stroke risk increases by 44% [28, 29].

A number of anesthetic factors likely contributed to the higher morbidity, mortality, and prognosis of AIS. GA is usually accomplished by the administration of inhaled anesthetic agents, which are associated with a higher risk of cerebral hypoperfusion and increased ischemic injury [30, 31]. Inhaled anesthetic agents, such as isoflurane, have been proved to induce cerebral vasodilation and steal blood flow from ischemic areas with poor autoregulation [32]. In addition, induction and recovery of GA are often associated with significant hemodynamic changes, including hypotension and rapid blood pressure fluctuations, which could aggravate the ischemic injury [33]. Many studies have reported that GA may be associated with poorer neurological outcomes after EVT for AIS. For instance, the meta-analyses by Brinjikji and his colleagues showed that patients with AIS undergoing EVT may have worse functional outcomes with GA compared with CS [11, 34]. In our meta-analysis, AIS patients in CS group showed the higher incidence of mRS score ≦ 2 at three months and decreased in-hospital mortality compared with these patients in GA group.

The main advantage of GA is decreased patient movement during EVT of AIS. Patients' movement during the procedure of EVT can compromise the safety and efficacy of the intervention and lead to wire perforation resulting in intracranial hemorrhage or vascular injury [35]. However, there were no differences among the rates of wire perforation, dissection, and hemorrhage between the GA and CS groups and thus the EVT procedure can be performed safely with CS according to the published studies [8, 15]. AIS patients treated with GA had a longer length of stay in the intensive care unit and hospital, higher risk of postoperative complications, and larger infarct volumes [15]. In this study, the utilization rates of IA rtPA and thrombolysis in the hospital between the two groups were similar, indicating that type of anesthesia would not impact the procedure of EVT.

Compared with the previous meta-analyses by Brinjikji and his colleagues [11, 34], the present study showed the similar results and conclusion with high-quality evidence from the following point of view. First of all, we searched the MEDLINE, EMBASE, and Cochrane Central Registers of Controlled Trials from inception to July 2017 to compare the effect of CS versus GA on the EVT in AIS patients. Second, we defined the incidence of mRS score ≦ 2 at hospital discharge and one or three months as the primary outcomes, suggesting good functional prognosis in AIS patients undergoing EVT. Third, we performed the trial sequential analysis to calculate the optimal event size requirement and GRADE evidence profile for each analyzed outcome.

However, there were several limitations and deficiencies in this study. The majority of included studies were retrospective observational studies resulting in moderate heterogeneity and quality evidence of analyzed outcomes, which was likely unreliable because of the small or incomparable number of available studies. Worse initial stroke severity could be prone to choose GA during EVT and contribute to the higher rates of posttreatment morbidity and mortality. Still, we did not analyze the outcomes on the basis of stroke location or complications during EVT. In fact, the previous studies had shown these outcomes between CS and GA groups.

In conclusion, this systematic review and meta-analysis of 13 studies and 2638 patients found that AIS patients receiving CS had significantly lower rates of mortality and good functional outcome (mRS ≦ 2) compared with GA patients. However, these findings are mainly based on the retrospective nonrandomized studies. Additional RCTs are needed to determine the differences in outcomes in AIS patients receiving GA or CS.

## Figures and Tables

**Table 1:** Characteristic and quality assessment of included studies in the meta-analysis.

Studies, year	Type of study	Type of stroke	Type of endovascular treatment	Number with CS versus GA	Selection	Comparability	Outcomes
Abou-Chebl et al. [8], 2010	Retrospective	AIS	Solitaire stent^a^	85/196	++++^b^		+
Davis et al. [12], 2012	Retrospective	AIS	IA rtPA and mechanical thrombectomy	48/48	++++	++	+
Hassan et al. [13], 2013	Retrospective	/	Endovascular procedure not specified	387/260	++++		+
John et al. [14], 2014	Retrospective	AIS	IA recanalization therapy	99/91	++++	++	+++
Jumaa et al. [15], 2010	Retrospective	Acute stroke	IA rtPA and mechanical thrombectomy	73/53	+++	+	+++
Just et al. [16], 2016	Retrospective cohort	AIS	IA rtPA, mechanical thrombectomy, thromboaspiration	67/42	++++	+	+++
Li et al. [17], 2014	Retrospective	AIS	IA rtPA, mechanical thrombectomy	74/35	++++	+	+++
Hendén et al. [18], 2017	RCT	AIS	IA rtPA	45/45	++++	++	+++
van den Berg et al. [9], 2015	Retrospective cohort	AIS	IA rtPA	278/70	++++		+++
Nichols et al. [19]; 2010	Pilot	AIS	IA rtPA	18/17	++++	++	+
Schönenberger et al. [10], 2016	RCT	AIS	Stent retriever or thrombus aspiration	77/73	++++	++	+++
Slezak et al. [20], 2017	Prospective	AIS	Solitaire stent or IA rtPA	135/266	++++		+++
Sugg et al. [21], 2010	Retrospective	AIS	Mechanical thrombectomy	57/9	++++		+

*Note*. CS: conscious sedation; GA: general anesthesia; AIS: acute ischemic stroke; IA: intra-arterial; RCT: randomized controlled trials. ^a^Covidien, Irvine, California. ^b^One plus indicates each quality item; 4 pluses are the maximum for the selection, 2 pluses are the maximum for the comparability, and 3 pluses are the maximum for completeness of the outcomes.

**Table 2:** GRADE evidence profile: consciousness sedation versus general anesthesia for patients hospitalized with AIS.

Outcomes	Quality assessment						
	Participants (studies), *n*	Median follow-up	Risk of bias	Inconsistency	Indirectness	Imprecision	Publication bias
mRS score ≦ 2	2648 (13)	3 months	No serious limitations	No serious limitations	No serious limitations	No serious limitations	Undetected
All-cause mortality	2552 (12)	In-hospital	No serious limitations	No serious limitations	No serious limitations	No serious limitations	Undetected
IA rtPA	1455 (6)	In-hospital	No serious limitations	No serious limitations	No serious limitations	No serious limitations	Undetected
Thrombolysis	697 (4)	In-hospital	No serious limitations	No serious limitations	No serious limitations	Serious limitations: small number of events	Undetected
